# Proposing UGV and UAV Systems for 3D Mapping of Orchard Environments

**DOI:** 10.3390/s22041571

**Published:** 2022-02-17

**Authors:** Aristotelis C. Tagarakis, Evangelia Filippou, Damianos Kalaitzidis, Lefteris Benos, Patrizia Busato, Dionysis Bochtis

**Affiliations:** 1 Institute for Bio-Economy and Agri-Technology (IBO), Centre for Research and Technology-Hellas (CERTH), 6th km Charilaou-Thermi Rd, GR 57001 Thessaloniki, Greece; e.filippou@certh.gr (E.F.); d.kalaitzidis@certh.gr (D.K.); e.benos@certh.gr (L.B.); 2 Department of Agriculture, Forestry and Food Science (DISAFA), University of Turin, Largo Braccini 2, 10095 Grugliasco, Italy; patrizia.busato@unito.it; 3 FarmB Digital Agriculture P.C., Doiranis 17, GR 54639 Thessaloniki, Greece

**Keywords:** smart agriculture, depth cameras, 3D mapping, 3D point clouds, situation awareness

## Abstract

During the last decades, consumer-grade RGB-D (red green blue-depth) cameras have gained popularity for several applications in agricultural environments. Interestingly, these cameras are used for spatial mapping that can serve for robot localization and navigation. Mapping the environment for targeted robotic applications in agricultural fields is a particularly challenging task, owing to the high spatial and temporal variability, the possible unfavorable light conditions, and the unpredictable nature of these environments. The aim of the present study was to investigate the use of RGB-D cameras and unmanned ground vehicle (UGV) for autonomously mapping the environment of commercial orchards as well as providing information about the tree height and canopy volume. The results from the ground-based mapping system were compared with the three-dimensional (3D) orthomosaics acquired by an unmanned aerial vehicle (UAV). Overall, both sensing methods led to similar height measurements, while the tree volume was more accurately calculated by RGB-D cameras, as the 3D point cloud captured by the ground system was far more detailed. Finally, fusion of the two datasets provided the most precise representation of the trees.

## 1. Introduction

### 1.1. General Context of RGB-Depth Cameras

Many tasks such as mapping, localization, navigation, 3D reconstruction of object, or scenery, among others, involve computer vision. Computer vision could be described as the technology that combines image processing through computational algorithms to obtain certain information from images [1,2,3] or vision systems utilizing laser scanners [4]. Focusing on the former case, a lot of studies have used RGB cameras so as to locate and distinguish the targets (e.g., fruits) from other objects by exploiting, for example, the shape, the color and the texture, usually combining their images with machine learning [3,5,6]. However, RGB cameras can only get two-dimensional (2D) information of the scene, while they are susceptible to variable light conditions and occlusions [7]. These challenges have been overcome through acquiring depth measurements of higher resolution, which have the potential to provide more detailed information about the scene. In particular, in the last decade, consumer-grade depth cameras have gained advantage over other sensors, given their low cost, portability, ease of use and measurement accuracy [8]. In brief, an RGB-D camera comprises two parts coupled together to give a dense matrix of pixel values; (a) an RGB camera for providing color information and (b) a depth camera for providing depth information [9]. Consequently, every pixel constructing the image is composed of color and distance values between a view-point and a certain point in the image (RGB-D values).

### 1.2. Use of RGB-D Cameras and Related Research in Agriculture

This type of camera has been applied in a number of areas of interest, such as indoor [10,11,12] and outdoor mapping [13], 3D reconstruction [14,15], motion and gesture recognition [16,17,18], and object detection [19]. Moreover, there has been an extensive use in robotics field and more specifically in navigation [19] and localization [2].

In recent years, stereoscopic vision depth cameras have been widely used in 3D reconstruction of objects and 3D mapping related to indoor environments [12,20]. Indicatively, the ZED stereo camera (Stereolabs Inc., San Francisco, CA, USA) has been employed in various indoor scenarios, namely volume designation of simple cubic and cylindrical objects through image segmentation process [21], crack detection and analysis on concrete surfaces using 3D data [22], terrestrial photogrammetry through an aerial mapping system [23], as well as the creation of indoor 3D mapping targeting to be used in studies for “smart” cities [24].

A plethora of researchers have studied the use of depth cameras in agricultural applications and identified their advantages and disadvantages in outdoor sceneries. An evaluation of five different depth cameras of three dissimilar technologies in agricultural applications was made by Condotta et al. [25]. According to their results, all cameras provided effective depth data indoors. Nonetheless, in outdoor environments the cameras using structured light and time-of-flight technology proved to be problematic, due to distortions by the intense lighting conditions. In particular, the aforementioned lighting may cause low contrast in the infrared image and lead to gaps in the corresponding depth image [25]. In outdoors applications, the most reliable data were provided by cameras using stereoscopy. Moreover, depth cameras were applied in agricultural applications for weed detection and above ground biomass volume estimation through 3D point clouds reconstruction [26]. In addition, efficient results in extraction of geometric structural parameters of vegetation with depth measurements were determined [27,28]. An effective approach of measuring the canopy structure on small plant populations in field conditions was presented in [29]. Furthermore, Jiang et al. [30] developed an approach to automatically quantify cotton canopy size in field conditions and showed the potential of using multidimensional traits as yield predictors. Additionally, an experiment using four different depth sensors in agricultural tasks was conducted by Vit and Shani [31], who estimated the quality of depth measurements for geometrical size estimation of agricultural objects with deep learning techniques. In tree crops, size estimation of mango fruits on trees in outdoor environment was made by Wang et al. [32].

Some of the RGB-D cameras are joined or can be combined with other sensors that allow for position and orientation of the camera to be recorded. Such sensors are, for example, inertial measurement units (IMUs) or global positioning system (GPS) sensors that provide positioning, velocity, and time information. As a result, depth cameras can be used to collect geometrical information about the environment and provide them as an input for robot localization and navigation. These added features in depth cameras are very useful for autonomous applications in agriculture or for capturing information about plants’ phenotype and growth. Studies have also been performed on the use of RGB-D cameras along with robotic systems to capture not only color information, but also spatial information about the environment. In addition, in [33] a solution was presented for autonomous obstacle avoidance performance of a UAV by using a deep learning-based object detection method and image processing with a depth camera. Another research, using depth camera mounted on an operational vehicle in an agricultural field, was presented in [34] for the reconstruction of the grapevines’ canopy to measure its volume as well as detect and count the grapevine bunches. Finally, Sa et al. [35] presented an accurate 3D detection method for sweet peppers peduncles in a farm field by using a depth camera on a robotic arm and a supervised machine learning approach.

### 1.3. 3D Mapping Procedures

With 3D mapping, the integration of appearance and shape information from depth sensors can be accomplished [36]. Some of the most commonly used tools for 3D mapping are the Octomap (University of Freiburg, Freiburg im Breisgau, Germany) [37] and real-time appearance-based mapping (RTABMap; Sherbrooke, QC, Canada). In particular, these tools are libraries related to the robot operating system (ROS).

The ROS software [38] for 3D mapping is widely used in robotic applications. More specifically, these tools can simultaneously capture and extract in a 3D map the environment area that a sensor is scanning, with the ability of representing it on a visualization tool. Octomap could be described as a probabilistic tool for 3D mapping, which is based on Octrees. An Octree is an information storing technique in a tree structure, in which there are nodes that each of them has eight “children”. The connection of all these nodes merges all the scanned data and generates continuous 3D maps. Apart from that, Octomap 3D mapping tool is a process which can efficiently recognize changes in the environment dynamically [39]. More specifically, the produced virtual environment with Octomap is composed of less noise from objects and robot position failures. Octomap meets four basic requirements. Firstly, free and occupied space, as it creates full 3D modeling and, secondly, it is updatable. Consequently, it is flexible, as the map can be expanded dynamically, while it is compact, as the produced map can be stored in memory and disk. It is worth mentioning that as compared with other 3D mapping tools, Octomap presents low computational load and memory usage. However, Octomap produces maps with only the depth data, which means that the points have only position information (*x*, *y*, *z*) and not color information (RGB).

Opposing Octomap, RTABMap creates 3D maps of the scanned environment with both color and depth data (RGB-D). A notable advantage of this 3D mapping tool is the fact that it provides a complete representation of the environment using the simultaneous localization and mapping (SLAM) algorithm. However, a main disadvantage of RTABMap is that it may lead to noisy maps, as it is unable to recognize dynamic objects [39]. In conclusion, these 3D maps can be stored for further processing and visualized in the ROS visualization tool (Rviz).

### 1.4. 3D Mapping Using Aerial-Based Systems

With the recent technological developments in the agricultural sector and the rise of digital agriculture and artificial intelligence, the use of unmanned aerial systems (UAS) is gaining popularity. This is mainly due to the fact that dedicated systems for commercial use have been made available to public [40]. Moreover, in contrast with satellite imagery, images acquired by UAS tend to demonstrate higher resolutions in both temporal (e.g., daily collections) and spatial (e.g., centimeters) level, while being insusceptible to cloud cover, thus, rendering them suitable for precision agriculture applications [41]. Furthermore, there is a high level of automatization in the analysis of the acquired images providing a range of products, such as orthomosaics with high spatial accuracy and 3D point clouds from the surveyed areas. An indicative recent study of using UAVs for agricultural applications is that of Christiansen et al. [42], where data collected from a LiDAR sensor mounted on a UAV were fused with global navigation satellite system (GNSS) and IMU data to carry out winter wheat field mapping for point clouds. Additionally, Anagnostis et al. [40] used UAS-derived images and deep learning to identify and segment tree canopies of orchards under diverse conditions. In addition, Gašparović et al. [43] combined classification algorithms with UAV images to map weeds in oat fields. Remarkably, RGB images from UAVs in conjunction with convolutional neural networks (CNNs) are constantly gaining ground [44,45,46], as highlighted in the recent literature review of Benos et al. [3].

### 1.5. Aim of the Present Study

All the above methods presented have certain drawbacks or do not consider RGB-D cameras. The aim of the present study was to investigate the use of RGB-D camera and UGV platform to autonomously map the environment of commercial orchards, map the location of trees and provide assessments of the tree size in terms of height and canopy volume by exporting and analyzing 3D point clouds. The results from the ground-based mapping system were compared with 3D orthomosaics acquired via an UAS. Finally, the fusion of the two datasets was performed as a means of reaching to a more accurate representation.

## 2. Materials and Methods

A ZED 2 depth camera, consisting of a stereo 2K camera with two color sensors (RGB) was used for the 3D reconstruction of orchard trees. The specific sensor has a horizontal field of view of 110° and can stream at a rate from 15 to 100 FPS, depending on the resolution. The camera’s connectivity is compatible to Universal Serial Bus (USB) 2.0. The baseline of 12 cm (distance between the left and right RGB sensor) manages a range of depth perception between 0.2 and 20 m. The most important characteristics of the ZED camera are summarized in Table 1.

The ZED 2 camera was connected to a NVIDIA Jetson TX2 development kit (NVIDIA Corporation, CA, U.S.A.), with Ubuntu GNU/Linux 18.04 (Canonical Ltd., London, UK) operating system. In this system, the ROS melodic distro was installed to access ROS tools, supporting the ZED 2 camera features. The Jetson TX2 processor was consisted of 8 GB of RAM, 32 GB Flash Storage, 2 Denver 64-bit CPUs, and Quad-Core A57 Complex. For the maximum speed and robustness of the system ensuring the best possible results, all GPU cores were set in full performance. The 3D reconstruction of trees was performed using the spatial mapping module of Stereolabs Software Development Kit (SDK) tool and RTABMap package of ROS. The SDK tool provides drivers for the camera, and several sample functions in Python programming language that were used for the measurements.

**Figure 1:**
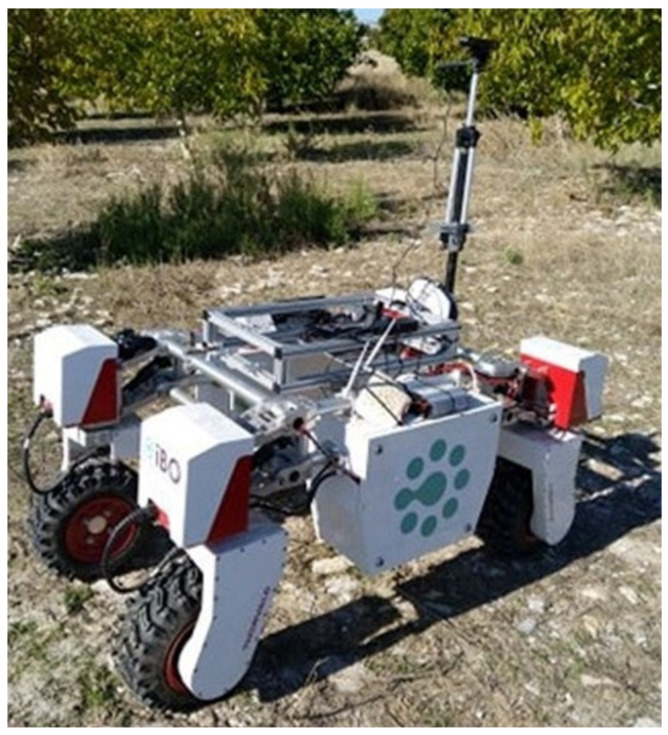
Setup of the ground-based scanning system mounted on Thorvald unmanned ground vehicle.

Due to the camera’s technology basic advantage of providing efficient results also in sunlight environments, this sensor is considered as an ideal solution for a robotic system operating outdoors. Moreover, the small size and compact structure of the camera makes it quite helpful to be used along with a robotic platform for applications such as mapping, object detection, etc. The sensing system was mounted on a Thorvald (SAGA Robotics SA, Oslo, Norway), which is an autonomous all terrain UGV (Figure 1) [47].

The Thorvald robotic vehicle was also equipped with a high accuracy GPS (RTK) as a means of providing the position of the robot and sequentially the position of the camera providing the ability to georeference the point cloud produced by scanning the orchard. Moreover, the scanning system was powered by the Thorvald’s battery. The setup was navigated in the field, capturing RGB-D images, collecting the necessary data to construct the 3D point cloud of the orchard.

The camera was located on a tripod attached on the Thorvald vehicle at about 1.5 m above the ground level facing sideways towards the trees canopy in horizontal position. In addition, the ZED camera was oriented towards the direction of the tree of interest, and it was manually adjusted as for the viewing angle and the height according to each tree. This adjustment was necessary due to variations in geometry characteristics of the canopy, volume and height of every individual tree.

The RGB-D-based scanning system setup was used in real field conditions to scan and construct the 3D representation of a commercial walnut orchard, located in Thessaly region, in Greece. The field measurements were conducted on sample trees of different height, volume and shape, on a sunny day during September 2020. The robot-camera system was used for in-field navigation, capturing RGB and depth data by steering a circle around each tree, at a distance of 2 m from the canopy, for about one minute. According to the acquisition rate, this procedure produced 3500 frames per sample tree. The camera readings were acquired at a high frame rate, namely 50–60 frames per second, providing sufficient overlapping among the frames for better 3D reconstruction of the model, as the SDK tool (Stereolabs) used for the 3D point cloud generation, merges the additional points of the scene and creates a more complete point cloud. The overlapped areas allow for 3D model construction by estimating the relative position of the camera for each frame. Several parameters of the sensor were adjusted through the SDK tool, such as brightness, saturation and contrast. Furthermore, according to the camera’s application programming interface (API) documentation, several parameters were set to fit in the field conditions. Specifically, the resolution of the camera was set to 1280 × 720 pixels (720p), and the point cloud mapping resolution was set to 2 cm. Additionally, the depth data range was set between 0.4- and 5-m distance from the camera position to create point cloud with high dense geometry and high resolution. Every point cloud of each tree was stored for further processing and saved in an object (OBJ) or polygon (PLY) file format, which is compatible with various point cloud and image processing software, such as Meshlab [48] and CloudCompare [49].

**Figure 2:**
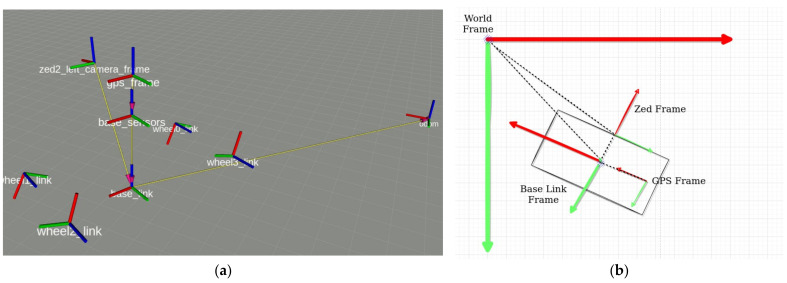
Location and orientation of the RGB-D camera in the relative coordinate system using the IMU (**a**) and georeferencing of the vehicle and the RGB-D to UTM coordinate system (**b**).

The ROS framework was utilized by the robotic platform to navigate in the field, while supporting data acquisition through the integrated “rosbag” tool. The system was recording simultaneously the RGB-D data from the ZED camera and the accurate position of the robotic vehicle utilizing the RTK-GNSS. The ZED camera uses its internal IMU to set the location and direction of the camera in relative coordinates. Therefore, it provides the RGB-D information in relative geodetic system. Combining the two datasets, the relative coordinates are referenced to the global coordinate system (GPS) “translated” into UTM coordinates (Figure 2). The “ros tf” library was utilized for this task.

The fusion of the two coordinate systems provided the ability to accurately georeference the spatial data captured by the camera which, after processing, produced the georeferenced 3D point cloud of the orchard corresponding to reality.

**Figure 3:**
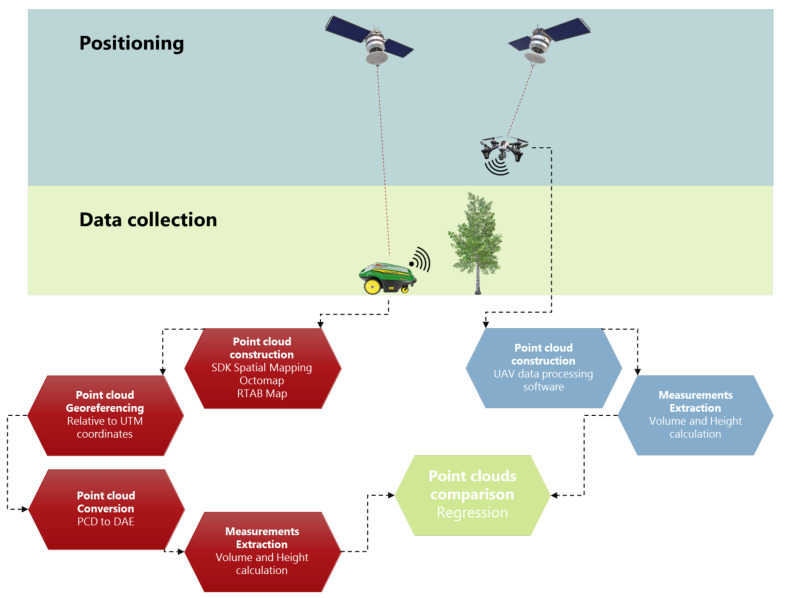
Flow chart of the procedure for the 3D point cloud construction using data from ZED 2 camera and its comparison with the UAV derived point cloud.

After the point cloud extraction, the height and volume of each tree was computed using CloudCompare and its internal tools. During this process, the point cloud is generalized to a surface elevation model and consequently the volume is calculated on the basis of the difference between a fixed ground elevation and the surface model. For the given case, the ground level was set as the lowest point for each tree point cloud. In other words, this technique is similar to draping fabric over the tree and computing the volume under the fabric. The resolution for the volume measurement was set equal to 2 cm. Furthermore, the density of points was calculated. The workflow of the data analysis followed in the study is briefly presented in Figure 3.

In addition to the ground-based scanning system, the orchard’s structure was also mapped from above using a UAV. The flight occurred during the same period with the ground-based measurements to ensure the comparability between the two-point cloud producing methods. The UAV was a quadcopter (Phantom 4, DJI Technology Co., Ltd., Shenzhen, China) equipped with high accuracy GNSS (real-time kinematic—RTK) and high-resolution RGB camera (5472 × 3648 resolution, at a 3:2 aspect ratio). The use of RTK GNSS was necessary in order to accurately geotag the acquired aerial images, while the flight plans were parametrized accordingly (UAV flight height, speed, number of captured images, side overlap, and forward overlap ratio) to produce high accuracy, below centimeter pixel size, orthomosaics. The produced orthomosaic can accurately provide the top view of the tree canopies in 2 dimensions and, thus, it was utilized as the ground truth for measuring the canopies’ surface. The 2D point cloud acquired by the ZED camera was compared with the orthomosaic.

For the purpose of comparing the measurements derived from the ground-based systems against those of aerial-based systems, simple linear regression analysis was utilized taking also into account the 95% confidence interval.

## 3. Results and Discussion

In order to estimate the true position of an object (a tree within the orchard in this case), the first step was to create a 3D model of the object in relative coordinates with the position of the camera as the axis origin and then set it in real-world coordinates by aligning this model to a known point (based on the camera position). This georeferenced point cloud aimed to be compared with a 3D point cloud produced from a UAV. Moreover, the georeferenced point cloud was imported in quantum geographic information system (Q GIS) to check the converted point cloud with a 2D georeferenced raster image of the same area. Reprojecting the point cloud in a real-world coordinate system provided the possibility to be used in various future simulation agricultural applications and robot tasks, such as object detection, spraying, or harvesting [50].

**Figure 4:**
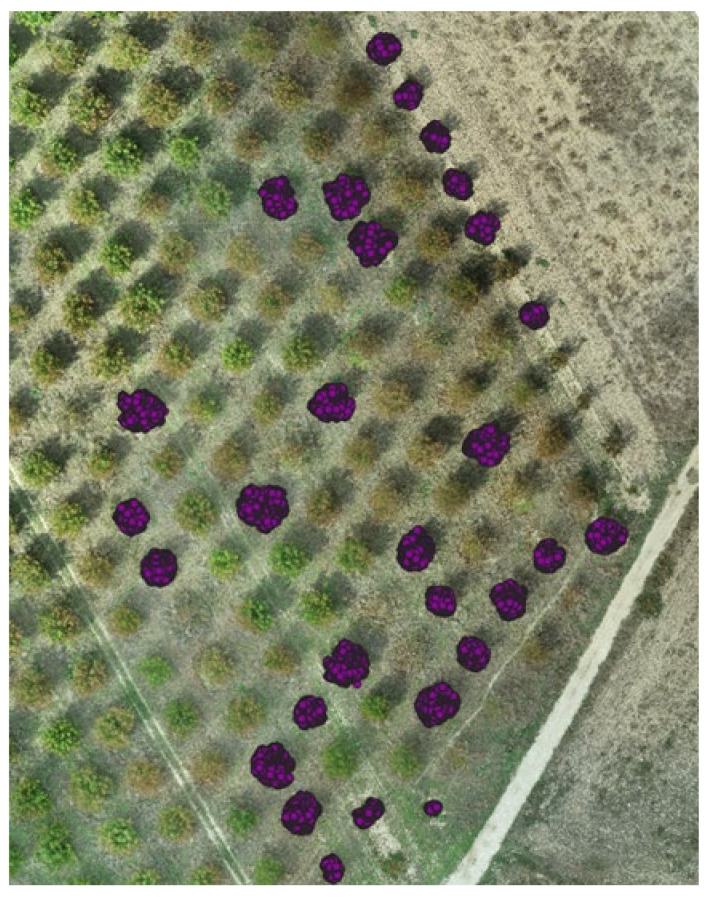
The projection of the point cloud (top view) of the orchard in 2D, mapped with the two methods used in the study; the ground-based system using depth camera mounted on Thorvald UGV and the orthomosaic exported from aerial images acquired using UAV.

**Figure 5:**
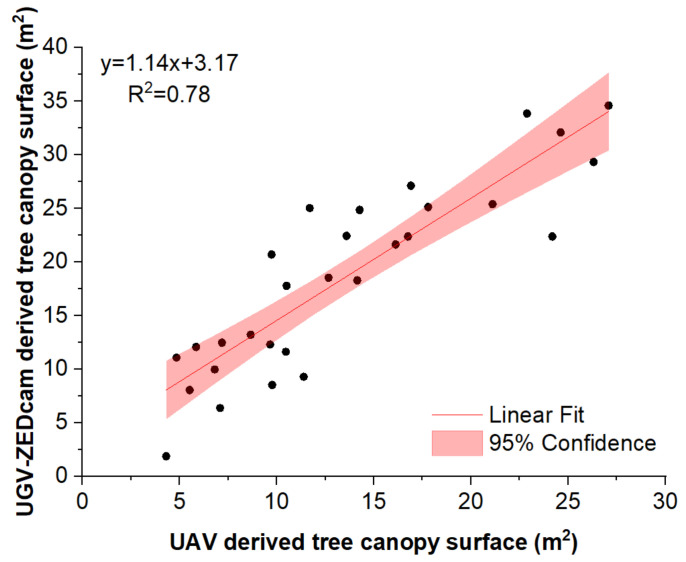
Comparison between the canopy size estimated by the ground-based and the aerial-based systems used in the study; the colored area shows the lower and upper confidence (95%) limits.

The representation of the orchard in two dimensions provided a general idea of the top view of the trees within the orchard, hence, providing the ability to estimate the canopy surface. This information can be valuable for estimating the age and the yield potential of each tree. In our study, the 2D representation also served as the first stage for the comparison of the two data acquisition methods. In Figure 4, the top view of the georeferenced point cloud acquired by the ground-based measuring system is projected overlayed on the detailed georeferenced orthomosaic constructed from the UAV-derived aerial images. The orchard, at the time of measuring, consisted of trees of different canopy size, color, and stage (fully developed, partly defoliated, or defoliated). Despite of the heterogeneity of the trees within the orchard, the results from the two methods were similar. This was also confirmed by the results of the regression between the two (Figure 5).

**Figure 6:**
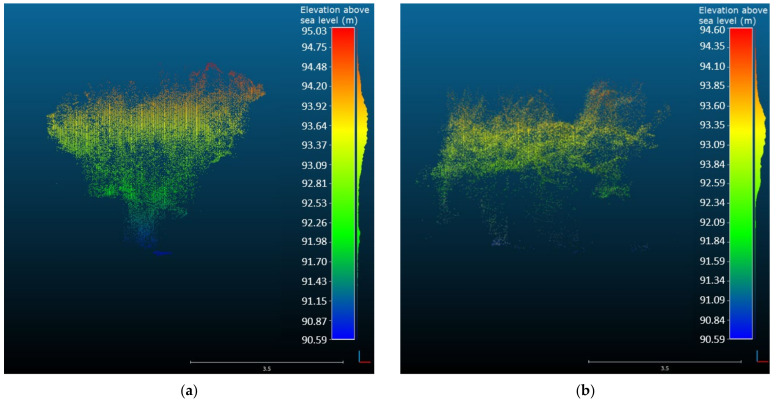
The side view of the georeferenced 3D point cloud of a sample tree captured by the ground-based system using the ZED camera (**a**) and by the UAV aerial-based system (**b**), used to estimate the tree height.

For the representation of the collected data in the three-dimensional world, the 3D point clouds were produced and converted to digital asset exchange (DAE) format, as to estimate the tree dimensional parameters, namely the height and canopy volume. Given that the datasets were georeferenced using high accuracy GNSS, the height of the captured trees could be accurately calculated (Figure 6). Furthermore, the robot-camera setup presented accurate results of the volume measurements of the trees confirming that the RGB-D cameras can serve as useful tools for agricultural applications, such as fertilizing and spraying, being part of decision support tools for variable rate applications according to the characteristics of each tree.

It is worth mentioning that the ZED camera, with the setup and adjustments used in the study, could not accurately detect the end details of the trees, such as thin branches, individual leaves, or nuts, as it could not provide extremely dense point clouds that are required for such tasks. Increasing the acquisition rate and the camera resolution and scanning more than one circles around each tree would enrich the point clouds producing very detailed point clouds. However, this would not be practical in agricultural applications, since it would be time consuming and hardware requirements for proper data acquisition and processing would significantly increase. In our system setup, despite the limitations, the constructed point cloud provided a model of the trees within the orchard very close to reality. This result is in agreement with the conclusions presented in [51], where the use of low-cost 3D sensors provided reliable results for plant phenotyping and can be applied in automated procedures for agricultural applications.

**Figure 7:**
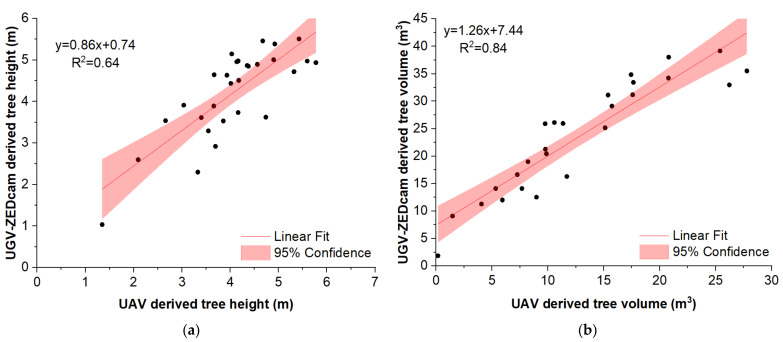
Comparison between the trees dimension measurements; trees height (**a**) and trees volume (**b**), derived by using the two sensing methods; the UAV aerial-based system and the UGV-ZED depth camera ground-based system; the colored areas show the lower and upper confidence (95%) limits.

Comparing the two capturing systems, the ZED camera provided a good representation of the trees, capturing details of the trunk, the lower, and mid canopy. Moreover, the center of the top canopy had some gaps due to the position and the viewing angle of the camera (Figure 6a). Conversely, the point cloud derived from the orthomosaic produced by the UAV aerial images provided a good representation of the top of the canopy, but had poor performance in the representation of the middle and lower canopy and the tree’s trunk (Figure 6b). This was expected, since by definition the UAVs can capture the top view of the objects, being unable to penetrate inside and under the canopy. However, some points of the lower canopy, the trunk, and the ground were captured making feasible the accurate estimation of the tree height, calculated by subtracting the ground surface elevation from the top of the canopy elevation. This fact led to similar height measurements from both measuring methods (Figure 7a).

From a practical point of view, a significant drawback of using the ZED camera ground-based system was the time needed to navigate within the orchard and capture the given number of trees. On the other hand, the aerial images derived from the UAV platform could be acquired within a short flight.

**Figure 8:**
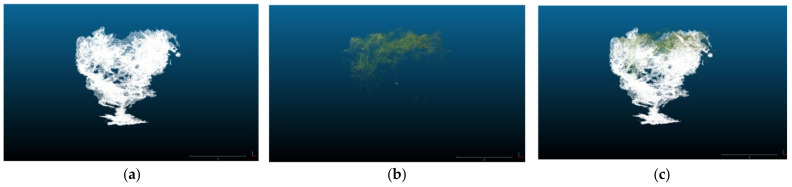
Point clouds of a sample tree derived by the UGV-ZED depth camera ground-based system (**a**), the UAV based aerial system (**b**), and the fusion of the two point clouds (**c**).

**Figure 9:**
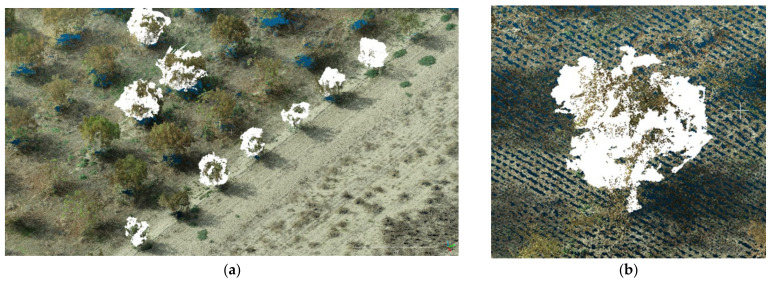
The 3D projection of the point clouds exported with the two methods used in the study; the ground-based system using depth camera mounted on Thorvald UGV ((**a**), white dots) and the orthomosaic exported from aerial images acquired using UAV ((**b**), colored dots).

In terms of tree volume measurements, the results from both methods showed similar trend; however, the UAV-derived tree volume was constantly lower by about 7.4 m^3^ while the slope of the relationship was 1.26 (Figure 7b). This is attributed to the fact that the UAVs can capture the upper part of the canopy, thus missing a significant part of the tree volume in the mid and lower canopy as seen in Figure 8b. However, these parts were captured in detail by the ZED camera. The latter managed to capture in detail almost the whole canopy, missing only a part of the middle top. As a consequence, the fusion of the two point clouds into a unified one constructed a more complete 3D model (Figure 8c and Figure 9).

The constructed point clouds can provide a useful input, by consequently converting them to meshes and importing in Gazebo simulation environment. The resulted virtual orchard environment may be used for testing of the robot navigation and localization. This testing will be carried out for estimation of the robot performance, in tasks such as autonomous navigation and obstacle avoidance before being evaluated in real field conditions. The visualization model in the Gazebo simulation environment can provide an adequate representation of the real orchard field and the possibility to make quality tests in a virtual world. In robotic applications, basic stage of the whole implementation is the algorithm testing part, which is performed in a virtual world before established in the real world. The use of simulation environments in various tasks and different environments, as to evaluate the robots’ performance, could be a quite costly and time effective procedure during the stage of testing and development of an application. As a result, using simulation environments in robotic applications, could optimize the robot behavior before the actual tests in the field [52].

## 4. Conclusions

In this study, the use of RGB-D camera to map the environment of commercial orchards was assessed and compared with 3D orthomosaics acquired using an UAS. The study verified that depth cameras, using stereoscopic vision to calculate the depth values, can provide accurate results in outdoor environments. The system, indeed, showed promising results, as it was capable to work under direct sunlight conditions capturing a high number of points with efficient resolution.

The produced point clouds provided efficient results for the structural parameters of the trees, as their shape and volume were adequately described. In some sample trees, lack of information of the inside and top of the tree canopy was observed. This limitation of the system was due to the initial settings of the camera’s parameters and/or due to the finite number of frames captured from each tree, set to the maximum of the hardware’s capabilities. Changes in these parameters or increasing of the image frames could possibly improve the 3D model reconstruction, though increasing significantly the processing time, hardware requirements, and, consequently, storage. Furthermore, scanning each tree more than once would significantly increase the point clouds’ density and accuracy, but this would affect the time required for the in-field scanning.

Overall, the UAV point cloud provided an accurate representation of the top view of the tree canopies. The orthomosaic, acquired by the RTK GNSS enabled UAS, was utilized as the ground truth for the 2D representation of the surface of the top view of the tree canopies. The 2D point cloud acquired by the ZED camera was successfully compared with the orthomosaic proving that the latter sensor can be an alternative providing accurate results. On the other hand, the point cloud from the ZED camera captured in much detail the structural characteristics of the trees all around, but had lack of information of the top canopy structure. Fusion of the two datasets led to construction of a more complete 3D model with increased accuracy providing a better representation of the tree structure. Focusing on the cost, aerial imaging is affordable, easier to operate and can cover larger areas as compared to on-ground systems. The RGB-D system on the other hand, may be facilitated with conventional agricultural machinery, capturing data while performing in-field operations, thus, minimizing the operational costs. Nevertheless, this study seeks to pave the ground to future applications following the trends of smart-autonomous farming leading towards Agriculture 4.0.

Finally, the 3D point clouds can be imported in Gazebo simulation environment to provide the virtual environment of the orchard to be used for efficient programming evaluation and demonstration of the robotic platform’s behavior and interaction in the orchard. Future developments include the automatization of the analysis procedure to provide the results in real time as the system navigates in the orchard. This will enhance situation awareness for safe and undisturbed navigation of the robotic platform in complex environments for the sake of avoiding possible injuries or damages [53]. In a broader perspective, further research is required towards improving the speed and accuracy of the existing cameras and image processing systems as well as decreasing the overall complexity [7,54,55,56]. Furthermore, fusion of data acquired by a group of unmanned vehicles could allow for better accuracy in a timely manner.

## Figures and Tables

**Table 1:** Main characteristics of ZED camera used in the study.

**Sensor**	RGB		
**Lens**	*f/*1.8 aperture		
**Depth range**	0.2–20 m		
**Field of view (horizontal, vertical, diagonal)**	110° (H), 70° (V), 120° (D)		
**Single image and depth resolution (pixels)**		Resolution (pixels)	Frame rate (Frames per second)
	HD2K	2208 × 1242	15 FPS
	HD1080	1920 × 1080	30/15 FPS
	HD720	1280 × 720	60/30/15 FPS
	VGA	672 × 376	100/60/30/15 FPS
**Complementary sensors**	Accelerometer, Gyroscope, Barometer, Magnetometer, Temperature sensor		
